# Alterations of Interhemispheric Functional Connectivity in Parkinson’s Disease With Depression: A Resting-State Functional MRI Study

**DOI:** 10.3389/fnhum.2020.00193

**Published:** 2020-06-05

**Authors:** Haiyan Liao, Jie Fan, Qin Shen, Sainan Cai, Min Wang, Chunyu Wang, Hainan Zhang, Jun Liu, Xiongzhao Zhu, Changlian Tan

**Affiliations:** ^1^Department of Radiology, The Second Xiangya Hospital, Central South University, Changsha, China; ^2^Medical Psychological Center, The Second Xiangya Hospital, Central South University, Changsha, China; ^3^Department of Neurology, The Second Xiangya Hospital, Central South University, Changsha, China

**Keywords:** voxel-mirrored homotopic connectivity, Parkinson’s disease, depression, resting state, functional connectivity

## Abstract

**Background:**

Depression is the most common non-motor symptom in patients with Parkinson’s disease (PD) with unknown mechanisms, but the diagnostic criteria of PD with depression (PDD) are not uniform.

**Purpose:**

The aim of the study was to investigate interhemispheric interactions between PDD patients and patients with PD without depression (PDND).

**Methods:**

The voxel-mirrored homotopic connectivity (VMHC) combined with the seed-based method was used to investigate intrinsic resting-state functional connectivity (RSFC) in 33 PDD patients, 60 PDND, and 47 healthy controls (HCs).

**Results:**

PDD patients exhibited a decreased VMHC in the bilateral medial frontal gyrus and paracentral lobule (MFG/PCL) than did PDND patients. Parkinson’s disease with depression had a decreased VMHC in the bilateral precentral gyrus than had PDND and HC (*p* < 0.05). Parkinson’s disease with depression had a decreased homotopic RSFC from the medial frontal gyrus (MFG)/PCL to the contralateral supplementary motor area (SMA) than had PDND (*p* < 0.05). The decreased homotopic RSFC from the right MFG/PCL to the left SMA was negatively correlated with Hamilton Depression Rating Scale scores (*p* < 0.05), but not with illness duration, Beck’s Depression Inventory, and Unified Parkinson’s Disease Rating Scale in PD patients.

**Conclusions:**

Our findings indicated that the occurrence of depression in Parkinson’s disease is associated with the dysfunctional connectivity from the MFG/PCL to the contralateral SMA, which could be used as potential neuroimaging markers for the diagnosis of depression in PD patients.

## Introduction

Depression is the most common and severe non-motor symptom in patients with Parkinson’s disease (PD), with a prevalence of around 30–50% (Ossowska and Lorenc-Koci, 2013). The incidence of PD with depression (PDD) increases with the progression of the disease (Rickards, 2005). It is commonly thought that depression is associated with reduced functioning and cognitive impairment; this is due to how such impairment may worsen motor and other non-motor symptoms, as well as the general quality of life in PD patients (Aarsland et al., 2011). Cumulative evidence suggests that PDD may be a consequence of the neurodegenerative process rather than simply a reaction to psychological distress (McDonald et al., 2003; Langston, 2006). Depression could be the initial symptom in PD (Postuma et al., 2012), associating with a fast decline of cognition and motor abilities (Ravina et al., 2007). However, the pathophysiological mechanisms of depression in PD patients are not fully understood. In addition, depression in PD patients is frequently unrecognized and untreated (Pachana et al., 2013) owing to the overlap of the signs and symptoms of depression with motor and other non-motor symptoms. Thus, a comparative study of brain structures and functions in PDD patients with and patients with PD without depression (PDND) could uncover the mechanisms of depression occurring in PD patients.

With the advanced neuroimaging approaches, many previous studies found that PDD patients have specific functional and structural abnormalities in the prefrontal, basal ganglia, and limbic regions. For example, studies on neural metabolic activity in the brain of PD patients showed abnormal metabolic activity in the frontal lobe, striatum, and subcortical or limbic regions including the thalamus, amygdala, hippocampus, anterior cingulate cortex, and insula in PDD patients (Huang et al., 2013; Vriend et al., 2014; Frosini et al., 2015; Qamhawi et al., 2015). Structural neuroimaging studies showed a gray matter loss and white matter reduction in the prefrontal, temporal, and some limbic regions (Matsui et al., 2007; Feldmann et al., 2008; Kostić et al., 2010). Studies on neural connectivity showed decreased connectivity in the bilateral anterior cingulate cortex, thalamus, and multiple tracts connecting to the left frontal and deep temporal lobes (Li et al., 2010; Huang et al., 2014).

Recently, resting-state functional magnetic resonance imaging (rs-fMRI) has been suggested as the most promising method to study the neural network mechanisms. However, most previous studies in PDD patients focus on regional deficits (Wen et al., 2013) and/or functional connectivity abnormalities of a specific network (Hu et al., 2015). In contrast, rare studies have focused on the integrity of the neural network. Voxel-mirrored homotopic connectivity (VMHC) is a data-driven voxel-based intrinsic functional connectivity (iFC) approach. Voxel-mirrored homotopic connectivity combines both the amplitude of low frequency fluctuation and regional homogeneity that can reflect the specific patterns of interhemispheric disconnection. Using this method, Zhu et al. (2016) found reduced VMHC values in the bilateral dorsolateral prefrontal cortex and calcarine cortex in PDD patients. However, Zhu et al. (2016) study did not use the seed-based iFC analysis.

We therefore hypothesized that PDD and PDND patients may have altered functional connectivity in some networks. In this study, we used a seed-based iFC analysis to reveal the heterotopic connectivity patterns in seed regions with altered VMHC in PDD patients. In addition, we also expected that VMHC measurements and resting-state functional connectivity (RSFC) would be clinically relevant, so the relationship between the altered VMHC values/RSFC and clinical variables was analyzed. This study can provide a more complete understanding on the abnormal interhemispheric synchronization in PDD and PDND patients.

## Materials and Methods

### Participants

Participants were recruited from The Second Xiangya Hospital, Central South University. Sixty-four PDND patients and 38 PDD patients were recruited from December 2015 to May 2019. Motor symptom was evaluated by the motor part of the Unified Parkinson’s Disease Rating Scale (UPDRS-III) and Hoehn and Yahr (H&Y) Scale. Parkinson’s disease with depression patients were diagnosed with *Statistical Manual of Mental Disorders*, Fifth Edition (DSM-V) by an experienced psychiatrist. The severity of depression was quantified with the 17-item Hamilton Depression Rating Scale (HDRS-17) and the 21-item Beck’s Depression Inventory (BDI). All participants underwent a semi-structured interview and were assessed with the Mini-Mental State Exam (MMSE). An MMSE score >17 for illiterate subjects, >20 for primary school literates, and >23 for junior high school and higher education literates were defined as a normal MMSE score in our patients. The inclusion criteria were as follows: (1) patient met the UK Parkinson’s Disease Society Brain Bank Criteria (Daniel and Lees, 1993); (2) patient was right-handed Han Chinese; (3) patient did not use anti-PD or antidepressant medications for 12 h; (4) patient had a motor symptoms of H&Y staging 1–3; (5) patient had a HDRS-17 score higher than 7 (PDD patients were diagnosed by DSM-V (Wang et al., 2018); and (6) patient had an MMSE score not lower than the corresponding education level (Li et al., 2016). Patients were excluded if they had (1) a history of head injury, stroke, or other neurologic or psychiatric disease; (2) abnormal MMSE scores; and (3) any disorders that may interfere with the assessment of the manifestation of PD. Fifty right-handed healthy Han Chinese with matched age, education, and sex were recruited for controls.

Detailed neuropsychological examinations including MMSE, HDRS-17, and BDI were used to exclude dementia and depression in healthy controls (HCs). Twelve participants (five PDD, four PDND, and three HCs) were excluded from analyses. This study was approved by Medical Research Ethical Committee of The Second Xiangya Hospital. Written informed consent was obtained from all participants prior to enrollment.

### Imaging Data Acquisition

Imaging data were acquired on a Siemens Skyra 3T MRI scanner at The Second Xiangya Hospital of Central South University. All participants were instructed to lie supine with eyes closed and no thinking but to avoid falling asleep. Their heads were fixed snugly with foam pads and straps to minimize head movement. Resting-state functional magnetic resonance imaging images were obtained using an echo planar imaging with 39 axial slices in 3.5-mm thickness, no gap, 2,500-ms repetition time, 25-ms echo time, 3.8 × 3.8 × 3.5-mm voxel size, 90° flip angle, 240-mm field of view, 64 × 64 data matrix, and 200 volumes. In addition, three dimensional T1-weighted magnetization-prepared rapid gradient echo sagittal images were acquired using the follow parameters: 176 slices, 1,900-ms repetition time, 2.01-ms echo time, 1.00-mm slice thickness, 1.0 × 1.0 × 1.0-mm voxel size, 9° flip angle, 900-ms inversion time, 256-mm field of view, and 256 × 256 matrix.

### Magnetic Resonance Imaging Data Preprocessing

The rs-fMRI data were analyzed using the Data Processing and Analysis of Brain Imaging (DPABI) v2.1 toolbox (Yan et al., 2016), based on the Statistical Parametric Mapping software (SPM12)^1^ for the MATLAB platform (The MathWorks Inc., Natick, MA, United States) as previously described (Zhou et al., 2018). Preprocessing comprised the following: (1) removal of the first 10 time points; (2) slice-time and motion corrections; (3) registration of the high-resolution individual T1-weighted images to the mean functional data; (4) segmentation of the high-resolution T1-weighted images with the Diffeomorphic Anatomical Registration Through Exponentiated Lie Algebra (DARTEL) toolkit and generation of a group template; (5) transformation and normalization of the resulting aligned data to the Montreal Neurological Institute (MNI) space with the segmented information from DARTEL; (6) resampling of the normalized rs-fMRI data to 3 × 3 × 3-mm voxels and spatial smoothing with an isotropic 6-mm full width at half maximum (FWHM) Gaussian kernel; (7) nuisance linear regression with the white matter, cerebrospinal fluid, and Friston-24 head motion parameters (including 6 head motion parameters and their historical effects as well as the 12 corresponding squared items) (Yan et al., 2013); and (8) removal of the linear trend and temporal bandpass (0.01–0.1 Hz) filtering. Finally, each subject was limited to a maximum displacement in any cardinal direction (*x*, *y*, *z*) of less than 1.5 mm and a maximum rotation (*x*, *y*, *z*) of less than 1.5° during each rs-fMRI scan. The group differences in head motion between the PDD patients, PDND patients, and the HC group were evaluated according to the published criteria (Van Dijk et al., 2012; Table 1). Six participants who displayed excessive head motion (translation >1.5 mm or rotation >1.5°) were excluded from further analysis.

**TABLE 1:** Demographic and clinical characteristics of the participants (mean ± SD).

**Variable**	**HC (*N* = 47)**	**PDD (*N* = 33)**	**PDND (*N* = 60)**	***p***
Gender (M/F)	20/27	15/18	35/25	0.224^a^
Age (years)	56.723 ± 8.554	56.455 ± 8.272	56.95 ± 9.883	0.969^b^
Education (years)	8.149 ± 3.73	7.091 ± 2.843	7.367 ± 3.361	0.334^b^
Disease duration (years)	NA	2.758 ± 2.634	2.304 ± 1.733	0.193^c^
MMSE	26.66 ± 3.385	26.788 ± 2.253	27.067 ± 2.449	0.744^b^
HDRS-17	2.686 ± 2.734	14.03 ± 4.536	3.214 ± 2.119	< 0.001^b^
BDI-21	9.31 ± 8.83	23.424 ± 10.666	8.286 ± 5.684	< 0.001^b^
UPDRS-III	NA	18.576 ± 12.523	14.800 ± 9.896	0.098^c^
H&Y	NA	1.652 ± 0.733	1.55 ± 0.637	0.256^c^
REL-GMV	0.408 ± 0.028	0.405 ± 0.026	0.403 ± 0.028	0.686^b^
REL-WMV	0.338 ± 0.021	0.337 ± 0.02	0.335 ± 0.024	0.727^b^
Mean Power_FD	0.096 ± 0.05	0.074 ± 0.037	0.09 ± 0.04	0.078^b^

*For comparisons of demographics: ^*a*^*p* value for the gender difference was obtained by chi-square test. ^*b*^*p* values were obtained by one-way analysis of variance (ANOVA) tests. ^*c*^*p* values were obtained by two-sample *t* test. PD, Parkinson’s disease; PDD, PD patients with depression; PDND, PD patients without depression; HC, healthy control; HDRS-17, 17-item Hamilton Depression Rating Scale; BDI-21, 21-item Beck’s Depression Inventory; UPDRS-III, Unified Parkinson’s Disease Rating Scale—motor part III; H&Y, Hoehn and Yahr Staging Scale; MMSE, Mini-Mental State Examination; REL-GMV, relative gray matter volume; REL-WMV, relative white matter volume; FD, framewise displacement; NA, not applicable.*

### Voxel-Mirrored Homotopic Connectivity

To obtain the interhemispheric connectivity at the single-voxel level, VMHC was performed using the DPABI toolkit. A group-specific symmetrical left–right hemisphere template was generated from the normalized T1 images of all subjects to minimize the geometric differences between hemispheres (Zuo et al., 2010). The same transformation of the T1-weighted images was then applied to the individual preprocessed resting-state functional images. For each subject, the homotopic connectivity coefficient was computed as the Pearson correlation between every pair of mirrored interhemispheric voxels’ time series. The resultant values constituted the VMHC and were used for group analyses.

### Seed-Based Intrinsic Functional Connectivity

We used a seed-region approach to evaluate iFC. To examine the specific networks that were influenced by homotopic connectivity, regions with altered VMHC among PDD, PDND, and HC groups were selected as regions of interest (ROIs) for seeds. Correlation functional analyses were performed by computing temporal correlation between each seed and the rest of brain in a voxel-wise manner. An entire brain *z* value map was created for each subject using the Fisher *r*-to-*z* transformation. A preceding united mask was used for whole-brain correction of multiple comparisons.

### Statistical Analyses

Data were analyzed using SPSS software (version 19.0; IBM, Armonk, NY, United States). One-way analyses of variance (ANOVAs) and *post hoc* two-sample *t* test in a pair-wise manner were used to identify the differential brain regions among the three groups with gender, age, and education as covariates. The clusters showing significant differences (voxel-level *p* < 0.01, cluster size >15 voxels, corresponding to a corrected *p* < 0.05 as determined by AlphaSim correction) in VMHC among three groups were extracted. The mean VMHC *z* values of these clusters were calculated. The Pearson correlation between the mean VMHC *z* values and the neuropsychological measurements was then calculated. Finally, the clusters with significant differences were used as seeds to perform a secondary seed-based FC analysis. The clusters showing significant differences in FC between PDD and PDND groups were extracted. The mean FC *z* values of these clusters were calculated. The Pearson correlation between the mean *z*-FC values and the neuropsychological measurements was analyzed. The mean FC values within these ROIs were further used for receiver operating characteristic (ROC) curves analyses. A cutoff was selected using Youden index and minimum distance from the coordinate (0, 1) on the ROC curve methods. All tests were two-tailed, and *p* < 0.05 was considered statistically significant.

## Results

### Sample Characteristics

Demographic and clinical characteristics of the participants are presented in Table 1. No significant differences in age, gender, education level, MMSE scores, relative gray matter volume, relative white matter volume, and mean framewise displacement were observed between any two of the three groups. Gender, age, and education level were further regressed, but no significant differences were observed. The relative gray matter volume and relative white matter volume used in the data analysis were measured from the segmented T1 images with CAT12 (A Computational Anatomy Toolbox for SPM 12). However, the HDRS-17 and the BDI scores were significantly different among the three groups (*p* < 0.001). There were no significant differences in PD duration, UPDRS-III, and H&Y staging between the PDD and PDND patients.

### Voxel-Mirrored Homotopic Connectivity Differences

**FIGURE 1:**
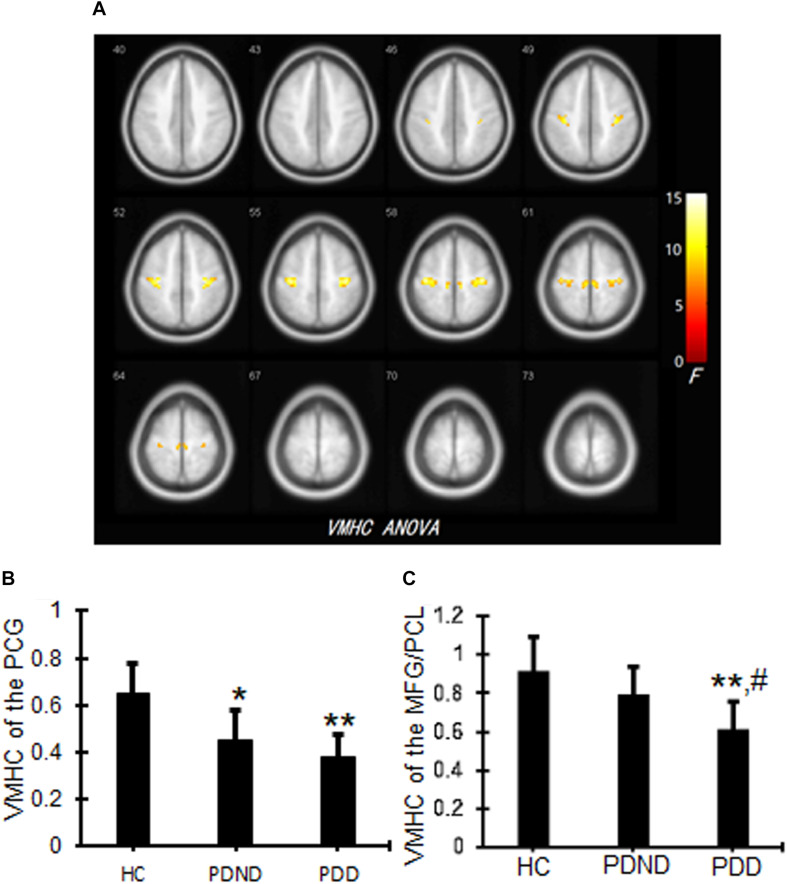
VMHC differences among PDD, PDND, and HC groups. The ANCOVA thresholds were set at a voxel-level *p* < 0.01, cluster size >15 voxels, corresponding to a corrected *p* < 0.05 as determined by AlphaSim correction. **(A)** Representative images. The yellow/red color bar indicates the *F* value from the ANCOVAs. **(B)** VMHC values of the PCG. *p < 0.05, **p < 0.01 vs. HC. **(C)** VMHC values of the MFG/PCL. Bar plots representing the mean VMHC values (and standard deviation) between three groups. **p < 0.01 vs. HC, ^#^*p* < 0.05 vs. PDND. VMHC, voxel-mirrored homotopic connectivity; PDD, Parkinson’s disease with depression; PDND, Parkinson’s disease without depression; HC, healthy control; ANCOVA, analysis of covariance; PCG, precentral gyrus; PCL, paracentral lobule.

Significant differences in the VMHC values of the bilateral precentral gyrus (PCG), medial frontal gyrus, and paracentral lobule (PCL) were observed among PDD, PDND, and HC groups (Figure 1A and Table 2). Voxel-mirrored homotopic connectivity in the PCG was significantly decreased in PDND compared with HCs, whereas VMHC in the PCG was notably decreased in PDD patients compared with HCs (Figure 1B and Table 2). Voxel-mirrored homotopic connectivity values in the bilateral medial frontal gyrus and PCL were significantly decreased in PDD compared with PDND and HCs, but there was no significant difference between PDND and HC groups (Figure 1C and Table 2).

**TABLE 2:** VMHC differences among PDD, PDND, and HCs.

**Brain region**	**Number of voxels (cluster size)**	**MNI coordinates of local maxima**			**Peak *z*/*t* value**
			
		***x***	***y***	***z***	
PCG	83	± 33	−27	45	14.56
MFG/PCL	16	± 9	−30	57	9.671
**HCs vs. PDD**					
Precentral (PCG)	83	± 33	−27	45	5.507
MFG/PCL	16	± 9	−30	57	4.167
**HCs vs. PDND**					
Precentral (PCG)	83	± 33	−27	45	4.199
**PDD vs. PDND**					
MFG/PCL	16	± 9	−30	57	−3.096

*PDD, PD patients with depression; VMHC, voxel-mirrored homotopic connectivity; PCG, precentral gyrus; MFG, medial frontal gyrus; PCL, paracentral lobule; PD, Parkinson’s disease; PDND, PD patients without depression; HCs, healthy controls; MNI, Montreal Neurological Institute.*

### Secondary Seed-Based Homotopic Resting-State Functional Connectivity

**FIGURE 2:**
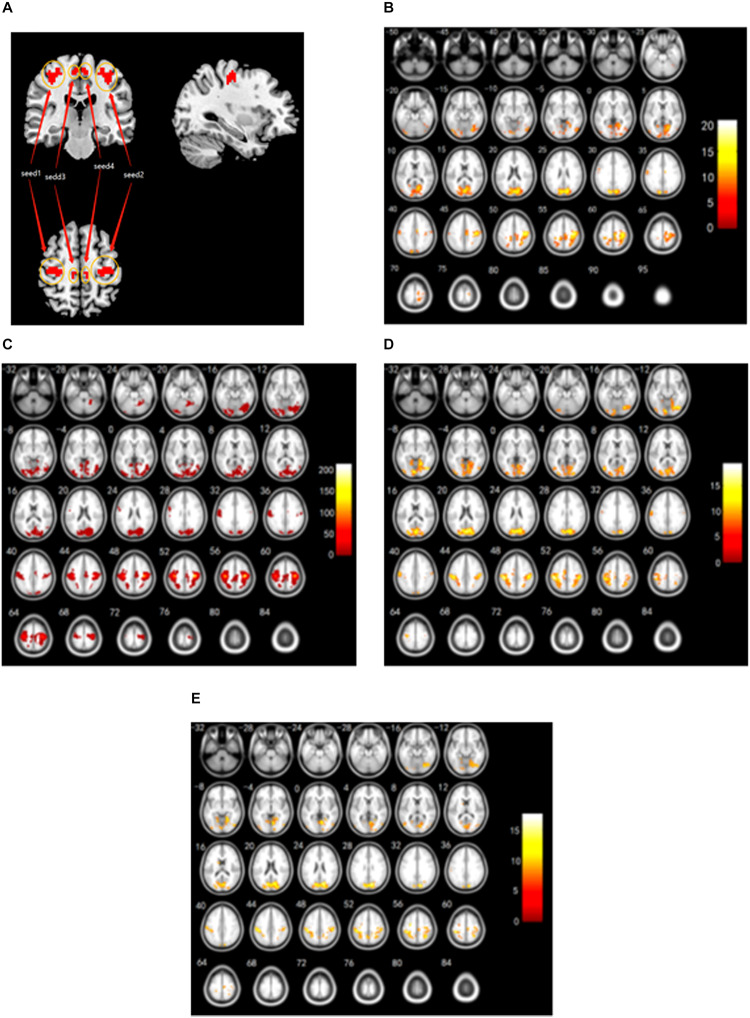
RSFC from the right/left PCG, and the right/left MFG/PCL seeds among PDD, PDND, and HC groups. **(A)** Seed regions with decreased voxel-mirrored homotopic connectivity. **(B)** RSFC patterns connected with the right PCG as the seed 1 from the entire brain among the three groups. **(C)** RSFC patterns connected with the left PCG as the seed 2 from the entire brain among the three groups. **(D)** RSFC patterns connected with the right MFG/PCL as the seed 3 from the entire brain among the three groups. **(E)** RSFC patterns connected with the left MFG/PCL as the seed 4 from the entire brain among the three groups. The color bars indicate the *F* values. RSFC, resting-state functional connectivity; PCG, precentral gyrus; PCL, paracentral lobule; PDD, Parkinson’s disease with depression; PDND, Parkinson’s disease without depression; HC, healthy control.

To evaluate the effects of disrupted interhemispheric connectivity on the relevant resting-state networks, the clusters involved in the right and left bilateral PCG and the right and left medial frontal gyrus (MFG)/PCL were used as the four ROI (including all the voxels in these clusters, Figure 2A) to investigate patterns of iFC throughout the entire brain among three groups. With the use of the right PCG as seed 1, a decrease in iFC was observed between the right PCG and the right fusiform gyrus, the left inferior occipital gyrus, the left lingual gyrus, the right calcarine fissure and surrounding cortex, and the bilateral postcentral gyrus (PoCG) among three groups (Figure 2B and Table 3) (AlphaSim corrected, combined with a different cluster size of >56 voxels).

**TABLE 3:** RSFC among PDD, PDND, and HC groups.

**Brain region**	**Cluster**	**Number of voxels (cluster size)**	**MNI coordinates of local maxima**			**Peak *z* value**
				
			***x***	***y***	***z***	
**Seed 1 (the right PCG)**						
Fusiform_R	1	209	27	−72	−15	12.452
Occipital_Inf_L	2	116	−39	−78	18	12.412
Lingual_L	3	62	−12	−81	−12	10.814
Calcarine_R	4	1,416	12	−78	27	17.95
Postcentral_L	5	62	−54	−18	42	11.132
Postcentral_R	6	1,286	36	−21	57	21.069
**Seed 2 (the left PCG)**						
Postcentral_L	1	5,382	36	−24	54	211.531
**Seed 3 (the right MFG/PCL)**						
Lingual_R	1	2,102	3	−81	21	18.117
Postcentral_L	2	676	−45	−30	48	19.309
Postcentral_R	3	361	45	−27	48	14.662
Supp_Motor_Area_L	4	80	−12	−18	54	12.056
**Seed 4 (the left MFG/PCL)**						
Lingual_L	1	31	−3	−84	−9	10.179
Occipital_Mid_L	2	66	−39	−90	−6	10.898
Cuneus_R	3	1,215	12	75	24	17.673
Lingual_L	4	10	−9	−78	0	8.314
Caudate_L	5	11	−9	3	15	10.09
Postcentral_L	6	376	−39	−36	57	16.459
Postcentral_R	7	280	33	−54	57	14.403
Precuneus_L	8	39	−3	−54	51	10.584
Precuneus_R	9	15	15	−45	51	9.583
Precentral_L	10	28	−30	−18	51	13.53
Supp_Motor_Area_R	11	21	6	−24	60	10.184

*RSFC, resting-state functional connectivity; PDD, Parkinson’s disease with depression; PDND, Parkinson’s disease without depression; HC, healthy control; L, left; R, right; PCG, precentral gyrus; MFG, medial frontal gyrus; PCL, paracentral lobule; MNI, Montreal Neurological Institute.*

With the use of the left PCG as seed 2, a significant difference in iFC was observed between the left PCG and the left PoCG among three groups (AlphaSim corrected, combined with a different cluster size of >74 voxels). Both PDD and PDND patients showed a decreased connectivity between seed 2 and the left PoCG compared with HCs (Figure 2C and Table 3).

With the use of the right MFG/PCL as seed 3, a decreased iFC was observed between the right MFG/PCL and the right lingual gyrus, and the bilateral PoCG, whereas an increased iFC between the right MFG/PCL and the left supplementary motor area (SMA) was observed among PDD, PDND, and HC groups (AlphaSim corrected, combined with a different cluster size of >49 voxels) (Figure 2D and Table 3). A decreased functional connectivity in the right MFG/PCL and the left SMA was observed in PDD patients compared with PDND patients. An increased functional connectivity from the right MFG/PCL to the left SMA was observed in PDND patients compared with HC.

With the use of the left MFG/PCL as seed 4, a decreased iFC was observed between the seed 4 and the bilateral lingual gyrus, the bilateral PoCG, the bilateral precuneus, the left middle occipital gyrus, the left caudate, the left PCG, the right cuneus, and the right SMA (AlphaSim corrected, combined with a different cluster size of >10 voxels) (Figure 2E and Table 3). A significant decrease in functional connectivity between the left MFG/PCL and the right SMA was observed in PDD patients compared with PDND patients.

### Relations Between the Voxel-Mirrored Homotopic Connectivity/Resting-State Functional Connectivity Values and Clinical Variables

PCG and MFG/PCL were selected as ROIs, because significant VMHC differences were observed among PDD, PDND, and HC groups. Linear correlation analysis was performed between the mean VMHC values within the ROIs and clinical variables in the PDD and PDND patients. No correlation was found between VMHC and illness duration, HDRS, BDI, and UPDRS-III in either PDD or PDND group. The clusters showing significant differences in seed-based FC between PDD and PDND groups were extracted for correlation analysis. The RSFC values of right MFG/PCL to the left SMA and RSFC values of the left MFG/PCL to the right SMA were then extracted. The decreased homotopic RSFC from the right MFG/PCL to the left SMA was negatively correlated with HDRS scores (*r* = –0.408, *p* = 0.012) in only PDD patients (Table 4).

**TABLE 4:** Correlations between the VMHC/RSFC and clinical variables.

**Clinical variables**	**VMHC**		**RSFC**	
	**PCG (*r/p*)**	**MFG/PCL (*r/p*)**	**Seed 3-cluster 4 (*r/p*)**	**Seed 4-cluster 11 (*r/p*)**
HDRS	−0.116/0.500	−0.113/0.511	−0.408/0.012	−0.316/0.073
BDI	0.124/0.471	0.256/0.132	−0.048/0.791	0.062/0.733
UPDRS-III	−0.128/0.459	−0.165/0.336	−0.339/0.054	−0.267/0.133
Disease duration	−0.11/0.552	0.058/0.734	0.208/0.245	0.289/0.103

*Seed 3-cluster 4 (the right MFG/PCL to the left SMA) RSFC; seed 4-cluster 11 (the left MFG/PCL to the right SMA) RSFC. VMHC, voxel mirrored homotopic connectivity; RSFC, resting-state functional connectivity; HDRS, Hamilton Depression Rating Scale; BDI, Beck’s Depression Inventory; UPDRS, Unified Parkinson’s Disease Rating Scale; PCG, precentral gyrus; MFG, medial frontal gyrus; PCL, paracentral lobule; SMA, supplementary motor area.*

### Discriminatory Analysis of the Resting-State Functional Connectivity From the Right Medial Frontal Gyrus/Paracentral Lobule to the Left Supplementary Motor Area and the Left Medial Frontal Gyrus/Paracentral Lobule to the Right Supplementary Motor Area

Because seed 3 (the right MFG/PCL to the left SMA) and seed 4 (the left MFG/PCL to the right SMA) exhibited significant RSFC differences between PDD and PDND groups, they were selected as ROIs. Mean RSFC values were extracted for further ROC analyses. The results showed that the area under the curve (AUC) of RSFC from seed 3-cluster 4 was 0.691 [95% confidence interval (CI): 0.569–0.814, *p* = 0.002] and the AUC of RSFC from seed 4-cluster 11 was 0.680 (95% CI: 0.563–0.798, *p* = 0.004). When separating PDD patients from PDND patients, the sensitivity and specificity for separating PDD from PDND were relatively high (Table 5).

**TABLE 5:** ROC analyses for PDD from PDND.

**RSFC values**	**AUC**	**Value**	**95% CI**	Cutoff point*	**Sensitivity**	**Specificity**
Seed 3-cluster 4	0.691	0.002	0.569–0.814	0.361^a^	0.967	0.394
Seed 4-cluster 11	0.680	0.004	0.563–0.798	0.343	0.767	0.576

Seed 3-cluster 4 (the right MFG/PCL to the left SMA) RSFC. Seed 4-cluster 11 (the left MFG/PCL to the right SMA) RSFC. PDD, Parkinson’s disease with depression; PDND, Parkinson’s disease without depression; RSFC, resting-state functional connectivity; ROC, receiver operating characteristic; AUC, area under the curve; CI, confidence interval; SMA, supplementary motor area. *By this cutoff point, the FC values of the seed 3-cluster 4 could correctly classify 32 of 33 PDD patients and 23 of 58 PDND patients, which resulted in a sensitivity of 96.7% and a specificity of 39.4%. The means of other cutoff points were similar.

## Discussion

To our knowledge, this is the first study that used the VMHC method combined with the seed-based homotopic RSFC to explore interhemispheric functional coordination in PDD and PDND patients. Using homotopic RSFC to measure interhemispheric synchrony or homotopy during the resting state is more important than other functional connectivity analyses (Gee et al., 2011). This study found that PDD patients had a decreased VMHC in the bilateral MFG/PCL; a decreased homotopic RSFC from the MFG/PCL to the contralateral SMA when compared with PD patients without depression PDND; and a decreased VMHC in the bilateral PCG when compared with PD patients without depression PDND and to HCs. The decreased homotopic RSFC from the right MFG/PCL to the left SMA was negatively correlated with the HDRS scores. The decreased RSFC in the right MFG/PCL to the left SMA could distinguish PDD patients from PDND.

The medial frontal gyrus (MFG) is located in the medial prefrontal cortex (mPFC). MFG is thought to be involved in cognitive regulation and affective processing (Hänsel and von Känel, 2008), whereas mPFC is widely known to play an important role in cognitive and limbic functions (Liang et al., 2015) and can modulate the expression of reward-seeking behavior through regulating the reward-related neural circuit (Ferenczi et al., 2016; Belujon and Grace, 2017). The abnormal spontaneous neural activity in the MFG has been demonstrated to be an important factor for the development of depressive symptoms in PD patients (Cardoso et al., 2009), whereas the right MFG activation is proposed as a biomarker for the occurrence and the severity of depression in PD patients (Zhi et al., 2019). Previous studies also observed a decreased activation in the mPFC of PDD patients compared with PDND patients (Cardoso et al., 2009). In contrast, the PCL controls motor and sensory innervation and plays an important role in somatosensation (Jellinger, 2008). Voxel-mirrored homotopic connectivity reflects iFC. This study found that PDD had a decreased VMHC in the bilateral MFG/PCL and a decreased homotopic RSFC from the MFG/PCL to the SMA compared with PDND. Taken together, the reduced VMHC and RSFC within the bilateral MFG/PCL may impede the down-regulation of the negative emotion and cause a deficiency in the reward-related neural circuit leading to anhedonia in PDD patients.

Our study showed that the decreased homotopic RSFC from the right MFG/PCL to the left SMA was negatively correlated with HDRS scores (*r* = -0.41, *p* = 0.02), but not with illness duration, BDI, and UPDRS-III in PD patients; this suggests that aberrant interactions between the right MFG/PCL and the left SMA are related to the severity of depression in PD patients. The results of ROC curve analyses indicated that the RSFC from the right MFG/PCL to the left SMA may have the ability to identify the PDD patients from PDND, which may reduce misdiagnosis. Thus, the disrupted right MFG/PCL–left SMA homotopic RSFC is not only related to the severity of depression in PD, but it can also distinguish PDD from PDND patients.

The midline core of the default mode network (DMN) comprises the posterior cingulate cortex, adjacent precuneus, and the mPFC (Sampaio et al., 2014). Functionally, DMN is involved in major human brain functions including spontaneous cognition (Xu et al., 2014). The motor network (MN) regions consist of the bilateral sensorimotor area, SMA, and bilateral cerebellum. Parkinson’s disease is traditionally considered as the most common movement disorder characterized by cardinal motor symptoms (Luo et al., 2014). However, in this study, PDD and PDND patients showed significant differences in homotopic RSFC from the MFG/PCL to the contralateral SMA. It could be said that disrupted RSFC in the midline structure of DMN to the MN can be used to distinguish PDD from PDND. However, using the bilateral PCG and the bilateral MFG/PCL for four seeds, we found a decreased homotopic RSFC from the right PCG to the bilateral PoCG, the left PCG to the left PoCG, the right MFG/PCL to the bilateral PoCG and the left SMA, the left MFG/PCL to the bilateral PoCG, and the left PCG and the right SMA among PDD, PDND, and HC groups. Overall, our findings suggest that PDD might depend on the damage to specific neural networks rather than on the dysfunction of single, discrete brain region.

We acknowledge several limitations of this study. First, our sample size is relatively small with the clinically heterogeneous group. Studies with a larger and clinically homogenous sample are needed to further validate our findings. Second, the human brain is not structurally symmetrical. Therefore, we tried to resolve this issue by smoothing the functional data and normalizing them to a symmetric template (Zuo et al., 2010). Hence, morphometric asymmetry could not account for the reduced VMHC (Kelly et al., 2011). Third, the current study mainly focused on the differences of interhemispheric synchrony between PD with and without depression PDND, only three neuropsychological tests (MMSE, HDRS-17, and BDI-21) were used, and we did not observe anxiety or apathy among the PD patients. A broader spectrum of tests may be helpful to evaluate the emotional and cognitive functions of PD patients. Fourth, the patients in the current study were not drug naïve. Although the anti-PD or antidepressant medications were stopped for 12 h before imaging and neuropsychological testing, the potentially confounding effects of chronic medications could not be avoided. Fifth, the lack of a primary depression group left the question of whether PDD shares a common neurobiological substrate with that of primary depression unanswered. Sixth, the multiple-comparison correction is not strict. Seventh, the seed areas were defined in the same sample that further analysis was conducted in. Finally, this is a cross-sectional study. It remains unsolved whether the found alterations represent state or trait characteristics of our patient cohort.

In conclusion, the occurrence of depression in PD might arise from the disturbance in the MFG and PCL, and disrupted RSFC in the midline structure of DMN to the MN can be used to diagnose depression in PD patients.

## Data Availability Statement

All datasets generated for this study are included in the article/supplementary material.

## Ethics Statement

The studies involving human participants were reviewed and approved by Medical Research Ethical Committee of The Second Xiangya Hospital, Central South University. The patients/participants provided their written informed consent to participate in this study.

## Author Contributions

CT and XZ contributed to the conception and design of the study. HL, SC, CW, HZ, and MW contributed to data collection. HL, JF, and QS contributed to data analysis. HL contributed to writing the manuscript. JL contributed to the English-language revision.

## Conflict of Interest

The authors declare that the research was conducted in the absence of any commercial or financial relationships that could be construed as a potential conflict of interest.

## Footnotes

1 http://www.fil.ion.ucl.ac.uk/spm/software/spm12/
